# AI‐Driven Fetal Liver Echotexture Analysis: A New Frontier in Predicting Neonatal Insulin Imbalance

**DOI:** 10.1002/jum.70053

**Published:** 2025-09-08

**Authors:** Karine S Da Correggio, Luís Otávio Santos, Felipe S Muylaert Barroso, Roberto N Galluzzo, Thiago Z L Chaves, Aldo von Wangenheim, Alexandre S C Onofre

**Affiliations:** ^1^ Division of Tocogynecology University Hospital Polydoro Ernani of São Thiago, Federal University of Santa Catarina (UFSC) Florianópolis Brazil; ^2^ Brazilian Institute for Digital Convergence, Technology Center Federal University of Santa Catarina (UFSC) Florianópolis Brazil; ^3^ Department of Clinical Analysis Federal University of Santa Catarina (UFSC) Florianópolis Brazil

**Keywords:** artificial intelligence, cord blood, C‐peptide, fetal liver, fetal ultrasonography, gestational diabetes

## Abstract

**Objectives:**

To evaluate the performance of artificial intelligence (AI)‐based models in predicting elevated neonatal insulin levels through fetal hepatic echotexture analysis.

**Methods:**

This diagnostic accuracy study analyzed ultrasound images of fetal livers from pregnancies between 37 and 42 weeks, including cases with and without gestational diabetes mellitus (GDM). Images were stored in Digital Imaging and Communications in Medicine (DICOM) format, annotated by experts, and converted to segmented masks after quality checks. A balanced dataset was created by randomly excluding overrepresented categories. Artificial intelligence classification models developed using the FastAI library—ResNet‐18, ResNet‐34, ResNet‐50, EfficientNet‐B0, and EfficientNet‐B7—were trained to detect elevated C‐peptide levels (>75th percentile) in umbilical cord blood at birth, based on fetal hepatic ultrasonographic images.

**Results:**

Out of 2339 ultrasound images, 606 were excluded due to poor quality, resulting in 1733 images analyzed. Elevated C‐peptide levels were observed in 34.3% of neonates. Among the 5 CNN models evaluated, EfficientNet‐B0 demonstrated the highest overall performance, achieving a sensitivity of 86.5%, specificity of 82.1%, positive predictive value (PPV) of 83.0%, negative predictive value (NPV) of 85.7%, accuracy of 84.3%, and an area under the ROC curve (AUC) of 0.83 in predicting elevated neonatal insulin levels through fetal hepatic echotexture analysis.

**Conclusion:**

AI‐based analysis of fetal liver echotexture via ultrasound effectively predicted elevated neonatal C‐peptide levels, offering a promising non‐invasive method for detecting insulin imbalance in newborns.

**Abbreviations**
AI: artificial intelligence
AUC: area under the ROC curve
BMI: body mass index
CNN: convolutional neural network
DICOM: Digital Imaging and Communications in Medicine
GDM: gestational diabetes mellitus
NAFLD: non‐alcoholic fatty liver disease
NICU: neonatal intensive care unit
NPV: negative predictive value
OGTT: oral glucose tolerance test
PPV: positive predictive value
ROC: receiver operating characteristic
T2DM: type 2 diabetes mellitus

Fetal hyperinsulinemia, characterized by elevated insulin levels, is a critical condition that can result in neonatal and long‐term metabolic complications, such as obesity, type 2 diabetes mellitus (T2DM), and cardiovascular diseases.^1^ Primary cause of fetal hyperinsulinemia is maternal hyperglycemia, particularly in conditions such as gestational diabetes mellitus (GDM), affecting 14–16% of pregnancies worldwide.^2^, ^3^

Emerging research suggests that fetal hyperinsulinemia is not limited to pregnancies with overt maternal hyperglycemia, such as GDM. Even when maternal glucose levels do not meet criteria for GDM, subtle variations in glycemia can still influence fetal insulin levels, indicating that a wider range of maternal metabolic factors may regulate fetal insulin production.^4^ Additionally, maternal obesity and altered lipid metabolism have been linked to increased neonatal insulin levels.^5^

Current diagnostic methods for assessing fetal metabolic disturbances are limited. Traditional approaches, such as maternal glucose testing and ultrasound imaging, are not sufficiently sensitive to detect subtle metabolic imbalances in fetus.^6^

Recent studies suggest that ultrasound fetal hepatic changes may provide valuable insights into metabolic disturbances caused by chronic hyperglycemia, which promote hepatic glycogen and lipid storage.^7^

Emerging image processing technologies enable detection of subtle tissue changes not visible to the naked eye.^8^, ^9^ Prior research on lung maturity has demonstrated the predictive value of such techniques for neonatal respiratory distress.^10^, ^11^ Given histological evidence of macrovesicular steatosis in livers of infants born to diabetic mothers,^12^ it is hypothesized that fetal hepatic echotexture could serve as a marker of poor glycolipid metabolic control. To achieve this, convolutional neural networks (CNNs) are employed for image analysis. CNNs, a type of deep learning algorithm, are ideal for medical image processing because they can automatically learn relevant features.^13^ By training on a large dataset of fetal liver images, the CNN model can detect patterns in echotexture that suggest impaired metabolic regulation.

This study aims to evaluate fetal hepatic echotexture as a predictor of neonatal high insulin levels by correlating ultrasound findings with cord blood C‐peptide, a marker of fetal insulin secretion, independent of maternal glycemic status. The choice of C‐peptide was made due to its greater stability and lower susceptibility to hemolysis compared to insulin.^14^

## Materials and Methods

### Study Design

This is a diagnostic accuracy study, conducted in accordance with STARD guidelines.^15^ The study analyzed the relationship between fetal liver echotexture—assessed through AI—and neonatal insulin levels, as indicated by C‐peptide concentrations in umbilical cord blood at birth. The research involved 2 groups of term pregnant women: 1 with GDM and the other without GDM, conducted at the University Hospital (HU‐UFSC) in Florianópolis, Brazil.

### Ethical Approval

The study received approval from the Research Ethics Committee (process number 5964191), and all participants women provided written informed consent for the use of their babies' prenatal images in future research.

### Sample and Inclusion/Exclusion Criteria

Pregnant women admitted for labor, induction, or elective cesarean section at the maternity emergency between September 2021 and September 2024 were invited to participate. A random sample was divided into 2 groups: Group 1, consisting of normoglycemic pregnant women (control group), and Group 2, consisting of pregnant women with GDM. GDM was defined according to the official guidelines of the Brazilian Society of Diabetes as either a fasting blood glucose level of 92 mg/dL or higher in the first trimester, or an abnormal result on the 75 g oral glucose tolerance test (OGTT) after 24 weeks.^16^

Based on an estimated 15% prevalence of GDM,^17^ 1381 live births in 2023, and allowing for up to 20% losses, a sample size of 161 participants was calculated with a 5% margin of error and a 95% confidence level. Inclusion criteria encompassed term pregnancies (≥37 weeks), participants aged ≥18 years, and agreement to participate through informed consent. Exclusion criteria included multiple pregnancies, pregestational diabetes, gestational age < 37 weeks, and comorbidities unrelated to GDM.

### Descriptive Variables

Clinical characteristics data included maternal age, ethnicity, parity, gestational age, and body mass index—BMI (calculated as weight/height^2^). Perinatal and neonatal outcomes, including delivery mode, the need for a first cesarean section, conditions at birth, such as newborns large for gestational age, Apgar score, respiratory distress, hypoglycemia, and neonatal intensive care unit (NICU) admission, were also investigated. Including a detailed description of the clinical and demographic characteristics is important because the diagnostic accuracy of a test can vary based on these factors, affecting the evaluation metrics. This clear description helps readers assess the relevance of the study to their clinical question.^15^

### Ultrasound

Ultrasound examinations, performed by experienced physicians using Siemens Acuson, Voluson 730, and Philips EPIQ Elite devices, measured traditional markers (eg, biparietal diameter, head and abdominal circumference, femur length, estimated fetal weight) as well as an innovative marker—fetal liver echotexture. This potential new marker was assessed using an average of 8.7 images per fetus in the axial plane of the fetal abdomen, a plane commonly used for measuring abdominal circumference, to evaluate fetal hepatic echotexture. A basic preset was used, with no post‐processing application, no zoom, and no use of calipers.

### Dataset

**Figure 1:**
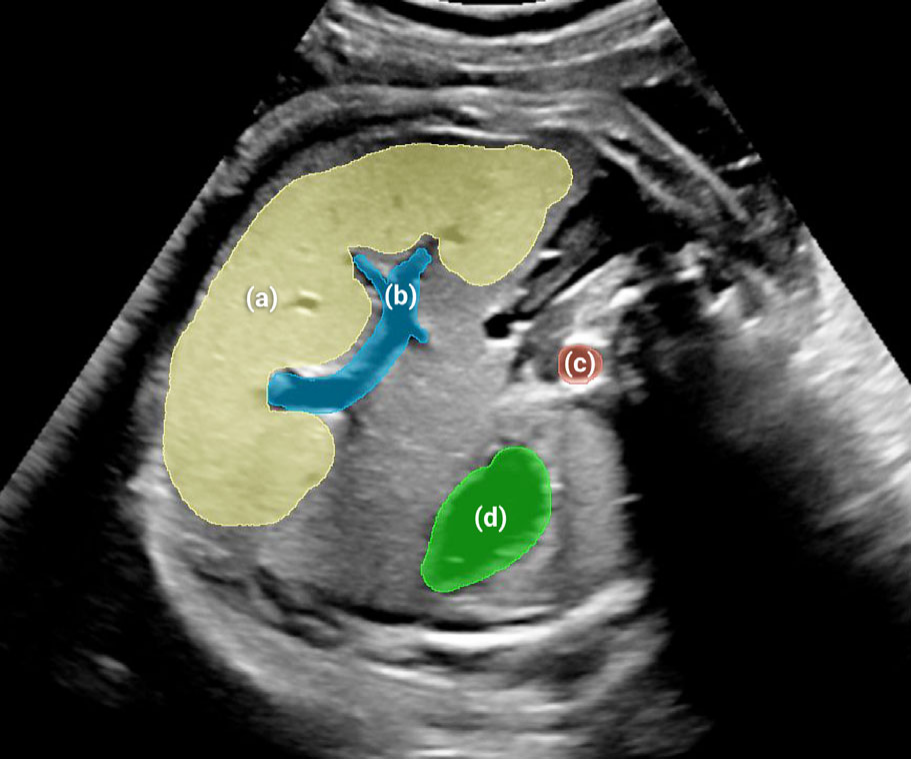
Traditional axial view of fetal abdominal circumference with liver annotation on ultrasound. (**A**) Liver, (**B**) intra‐hepatic portion of umbilical vein, (**C**) aorta, (**D**) stomach. These structures were annotated together in a parallel study about automated semantic segmentation and object detection, for which the dataset is available.^20^

The ultrasound images were originally saved in DICOM (Digital Imaging and Communications in Medicine) format in a secure hospital cloud, where they were subsequently downloaded and stored in a dedicated folder. Fetal abdomen images were selected using Radiant® (a DICOM viewer)^18^ and organized into patient‐specific folders. These folders were then opened in 3D Slicer®^19^ for experts to annotate the fetal livers, with the annotations saved as masks in NRRD format. The liver annotations, performed manually, consisted of the largest area of tissue identified in the axial plane, avoiding the hepatic portion of the umbilical vein, as shown in Figure 1A. Other structures (B, intra‐hepatic portion of umbilical vein; C, aorta; D, stomach) were annotated together in a parallel study about automated semantic segmentation and object detection, for which the dataset is also available.^20^

At this stage, the images had already been anonymized. A total of 2339 ultrasound images and their respective masks were initially included. During quality control, 606 images were discarded due to the presence of calipers, obvious blurring, or significant loss of image quality during conversion from DICOM to PNG format.

**Figure 2:**
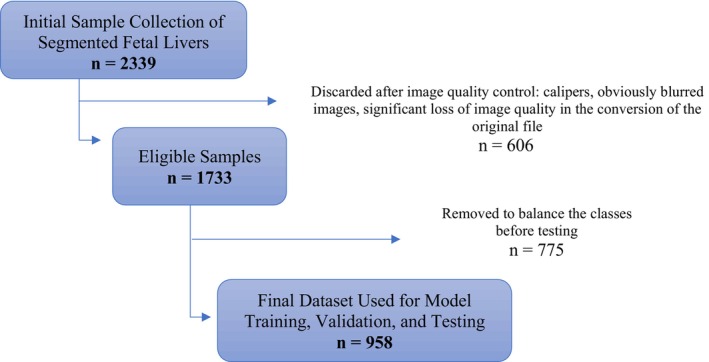
Flowchart of the selection process for fetal liver ultrasound images obtained from axial abdominal views in 325 term fetuses.

After this step, 1733 high‐quality images with correctly applied segmentation masks were retained. To achieve a balanced dataset, images were randomly removed from the overrepresented category until equal numbers of normal and altered fetal liver images were reached. The final dataset used in the analysis consisted of 958 images, as shown in Figure 2.

**Figure 3:**
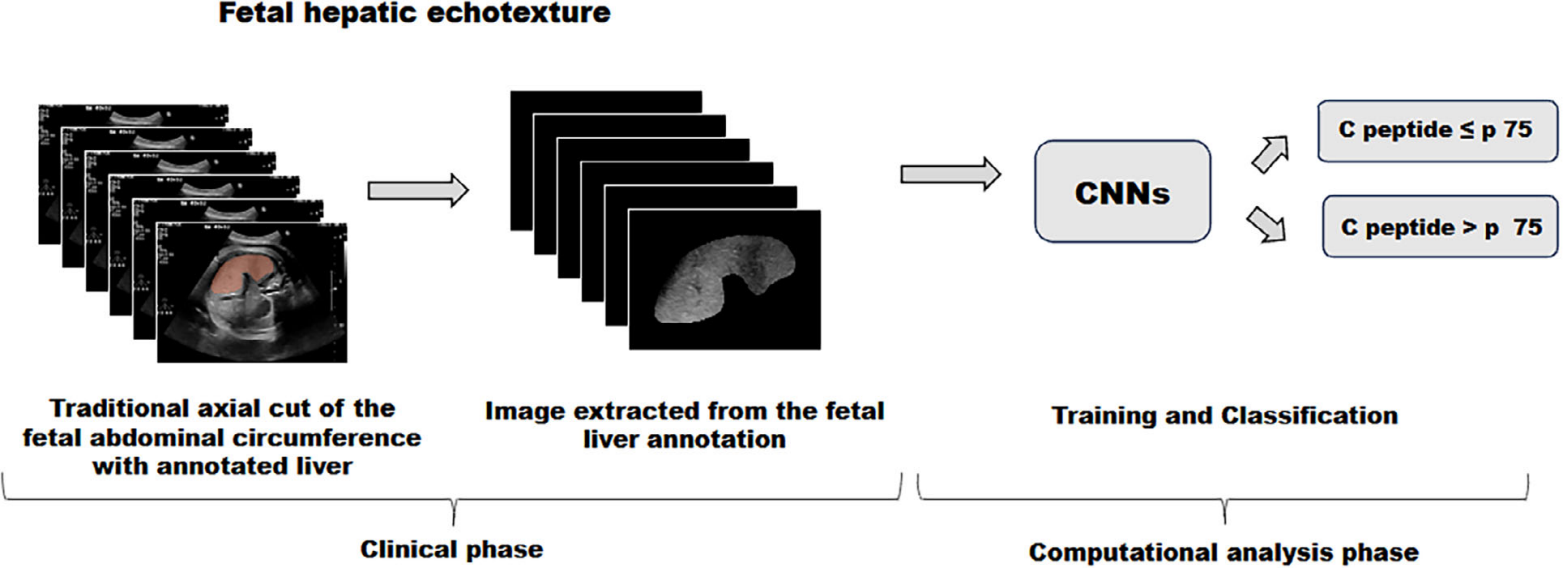
Overview of fetal liver image processing with the Neural Network. CNN, convolutional neural network; *p*, percentile. Images from the study dataset.

The fetal hepatic region of interest, obtained by the process previously detailed, was analyzed using AI classification models implemented using Python language libraries, these being deep learning frameworks built on PyTorch that facilitate rapid prototyping and efficient model training.^21^ The analysis used established CNN architectures, both from the ResNet and EfficientNet types. From ResNet, the ones used were ResNet‐18, ResNet‐34, and ResNet‐50. These architectures employ residual connections that address the vanishing gradient problem, enabling deeper networks to extract complex features from ultrasound images more effectively.^22^ For the EfficientNet side, EfficientNet‐B0 and EfficientNet‐B7 were employed. These models utilize a compound scaling method that uniformly adjusts network depth, width, and resolution to optimize performance while maintaining computational efficiency.^23^ All models were trained on the same annotated dataset to predict elevated neonatal insulin levels based on subtle variations in fetal hepatic echotexture. For each CNN model, pretrained weights were loaded. Mixed precision training was enabled to speed up computation. The learning rate was determined automatically using FastAI's learning rate finder. The model then was trained using the 1‐cycle policy over a maximum of 1500 epochs, with weight decay and gradient accumulation to stabilize the training process. Early stopping was employed based on validation loss, and the best model was saved during training. Albeit the existing literature about the topic of this study is very sparse, the existing one is enough in demonstrating that deep learning is a venue of interest for medical image analysis, including ultrasound images.^24^, ^25^ The steps of processing the fetal liver images for neural network training can be summarized in Figure 3.

Configurations used were kept consistent across all models, except for model‐specific details. Functions for saving data for comparison were identical for all of them. The dataset used for training was the same for all models and underwent preprocessing to ensure quality and balance, including systematic filtering to retain only valid fetal liver images and balanced classes for robust model performance. The training and validation sets were separated using an 80/20 split; in this manner, the models were never exposed to the validation dataset during training. The 80 percent for training was further divided with a 70/30 split between training itself and validation, this done per training epoch. Each model architecture was trained and fine‐tuned using mixed precision training and data augmentations to maximize prediction accuracy. To ensure that the training proceeded as expected and that the maximum possible accuracy was achieved, the metrics of accuracy, Dice, and precision were evaluated throughout the process. Models were trained using 1‐cycle learning rate policies, with early stopping and model checkpointing implemented to prevent overfitting.

### C‐Peptide

After delivery, 5 to 10 mL of umbilical venous blood was collected to measure C‐peptide and glucose levels using the ADVIA Centaur® system. Neonatal insulin levels were assessed by C‐peptide, with values above the 75th percentile in the normoglycemic group considered altered. These levels were correlated with fetal liver echotexture, which was evaluated using AI for computational analysis. By relying on objective, laboratory‐based variables (C‐peptide levels in cord blood) and AI‐driven assessment of hepatic echotexture, any potential biases were minimized.

**Figure 4:**
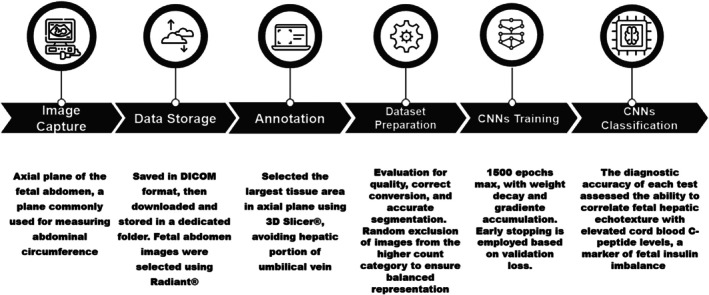
Infographic of the workflow, showing the entire process from image capture to final classification of individual fetal livers. Icons licensed by SlideBox.

Finally, all the methodology processes, showing the entire workflow from image capture to final classification of individual fetal livers as demonstrated in Figure 4.

### Statistical Analysis

Normality was assessed using the Shapiro–Wilk test. Numerical variables were analyzed through central tendency and dispersion measures, and categorical variables through absolute and relative frequencies. Group differences were evaluated using *t*‐tests, Pearson's chi‐square test, and Fisher's exact test when indicated. Diagnostic performance metrics, including sensitivity, specificity, positive predictive value (PPV), negative predictive value (NPV), and accuracy, were used to assess the test's ability to correctly identify true positive and true negative cases, as well as its overall diagnostic accuracy. The diagnostic accuracy test assessed the ability to correlate fetal hepatic echotexture with elevated cord blood C‐peptide levels, a marker of fetal insulin secretion, independent of maternal glycemic status.

## Results

A total of 2,339 ultrasound images, obtained from 325 different pregnancies (163 with GDM and 162 with normoglycemic pregnancies), were analyzed in this study. After assessing images for quality criteria, 606 images (25.9%) were excluded due to conversion problems with files, leaving a total of 1733 images for further analysis, as shown in Figure 2. To balance the classes before testing, 775 samples were removed, resulting in a final dataset of 958 images. Cord blood was collected from 271 newborns at birth (83.4%), and C‐peptide was analyzed. Baseline characteristics of the study population are presented in Table 1, while perinatal outcomes of included pregnancies are shown in Table 2. The GDM group was slightly older than the normoglycemic group, with more than half being multiparous and having a higher BMI compared to the non‐GDM group. The mean GA at delivery was higher in the normoglycemic group compared to the GDM group. In contrast, mean birth weight was similar between the 2 groups, although an AC above the 75th percentile was nearly 3 times more frequent, and the incidence of LGA neonates was twice as high in the GDM group.

**Table 1:** Clinical Characteristics of the Women Included in the Study

Variables	Group		*P* Value
	Normoglycemic (n = 162)	GDM (n = 163)	
Maternal age (years)	27.8 ± 6.6	29.5 ± 6.3	.01^a^
BMI (kg/m^2^)			
Preconception BMI	26.4 ± 5.4	29.4 ± 6.5	.002^a^
BMI categories			.003^a^
Thinness	4 (2.5)	1 (0.6)	
Normal	67 (41.4)	44 (27.0)	
Overweight	53 (32.7)	51 (31.3)	
Obesity class I	24 (14.8)	31 (19.0)	
Obesity class II	11 (6.8)	24 (14.7)	
Obesity class III	3 (1.9)	12 (7.4)	
Ethnicity			.54
Caucasian	125 (77.2)	121 (74.2)	
Non‐Caucasian	37 (22.8)	42 (25.8)	
Nuliparity			.04^a^
No	63 (38.9)	82 (50.3)	
Yes	99 (61.1)	81 (49.7)	

Values expressed as mean, standard deviation, absolute and relative frequency.

BMI, body mass index; GDM, gestational diabetes mellitus.

^a^ Statistically significant.

**Table 2:** Perinatal and Neonatal Outcomes of Newborns Included in the Study

Variables	Group		
	Control (n = 162)	GDM (n = 163)	*P* Value
Gestational age (days)	280.6 ± 7.8	272.9 ± 9.0	.002^a^
Mode of delivery			.92
Vaginal	93/161 (57.8)	95 (58.3)	
C‐section	68/161 (42.2)	68 (41.7)	
First C‐section	43/161 (26.7)	41 (25.2)	.93
US AC, mm	342.3 ± 19.5	347.2 ± 20.0	.03^a^
US AC > 75th percentile	23/158 (14.6)	65/161 (40.5)	<.001^a^
Birthweight, g	3424.1 (±435.9)^b^	3435.7 (±394.0)	.80
Relevant condition at birth			
LGA	10/161 (6.2)	20 (12.3)	.03^a^
Apgar at 1 minute <7	7/161 (4.4)	16 (9.8)	.16
Apgar at 5 minute <7	0/161 (0)	2 (1.2)	.37
Respiratory distress	8/161 (5.0)	17 (10.4)	.21
Hypoglycemia	4/161 (2.5)	22 (13.5)	.21
NICU admission	5/161 (3.1)	10 (6.1)	.20
Cord blood			
C‐peptide, ng/mL	0.9 ± 0.5^c^	1.1 ± 0.7^d^	<.01^a^
C‐peptide >75th percentile	355/132 (26.56.5)	58/139 (41.7)	<.01^a^
Glycemia	79.6 ± 26.5^e^	87.3 ± 35.8^f^	.05^a^

Values expressed as mean, standard deviation, absolute and relative frequency.

LGA, large for gestational age; NICU, neonatal intensive care unit; US AC, fetal abdominal ultrasound circumference.

^a^ Statistically significant.

^b^ n = 161.

^c^ n = 132.

^d^ n = 139.

^e^ n = 126.

^f^ n = 136.

Respiratory distress occurred in 25 cases and hypoglycemia in 26, mostly in the GDM group. Cord blood C‐peptide values >1.6 were considered above the 75th percentile, according to the reference curve constructed for the euglycemic group. 41.8% of the GDM group had C‐peptide levels >75th percentile. Glucose levels followed the same distribution, being higher in the GDM group.

**Figure 5:**
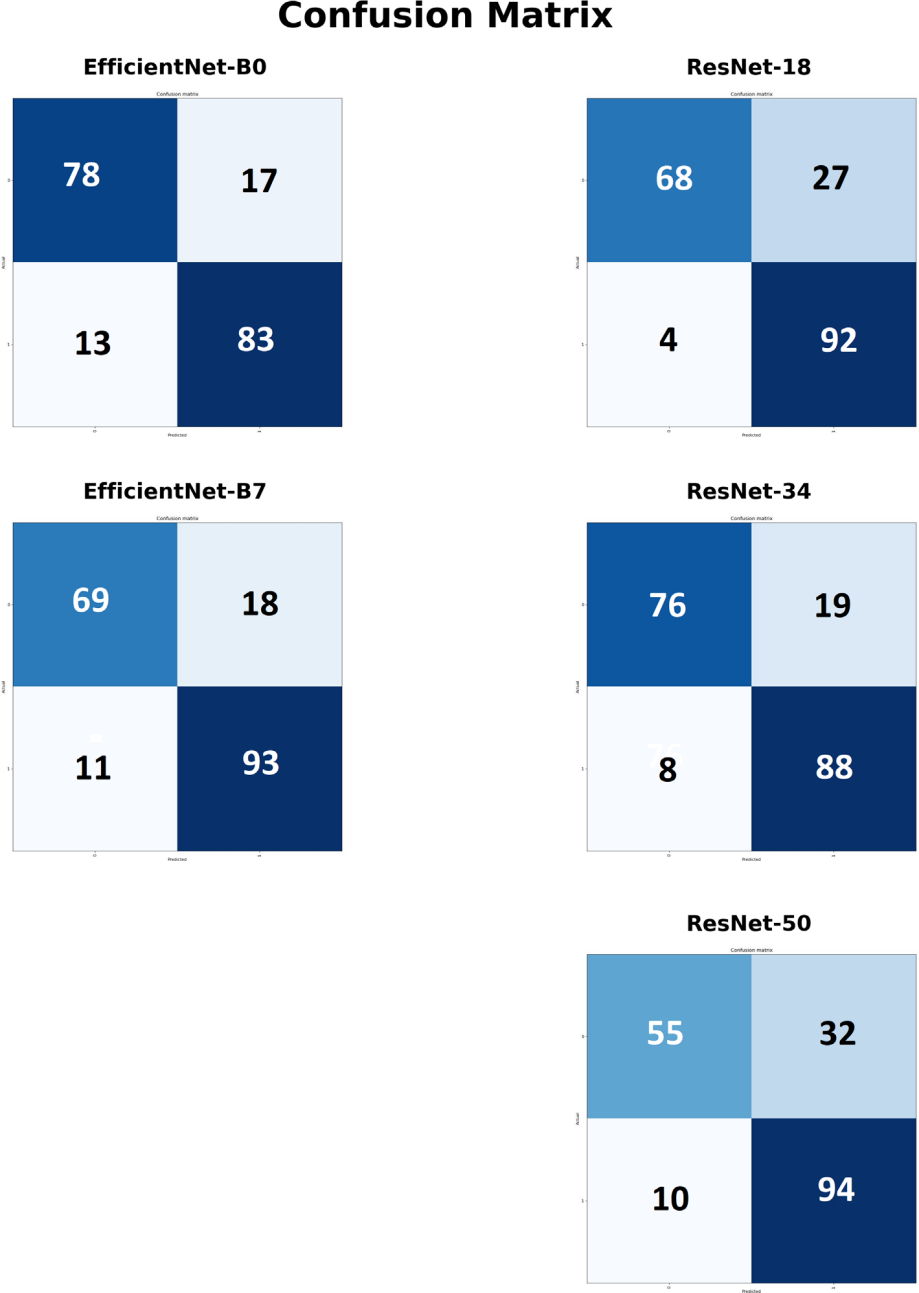
Confusion Matrices of the 5 CNN models. Confusion matrices of the 5 CNN models evaluated for predicting elevated insulin levels in umbilical cord blood based on fetal liver ultrasound texture. Each matrix displays the number of true positives (TP), false positives (FP), true negatives (TN), and false negatives (FN) for the respective model.

**Figure 6:**
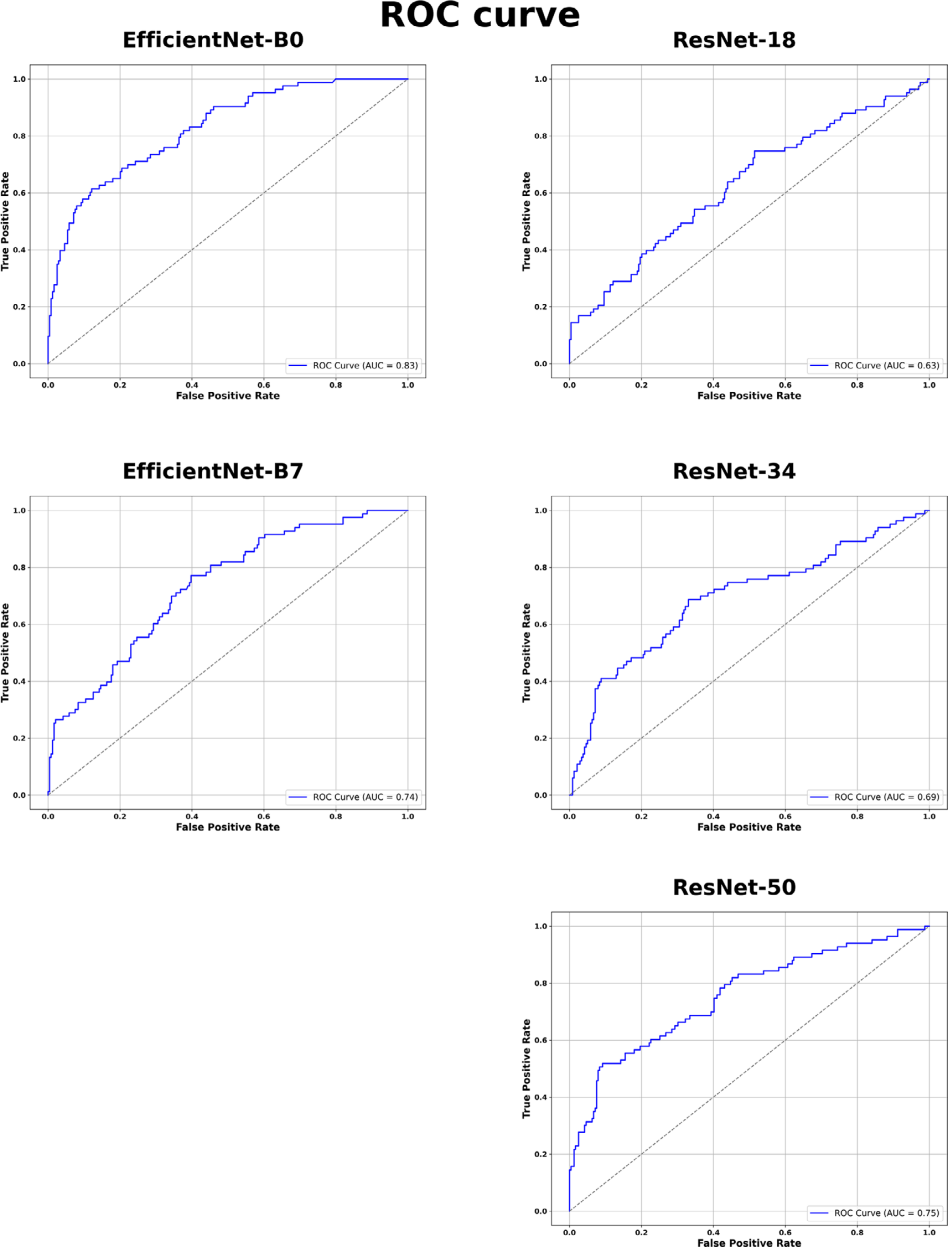
Performance ROC curves of 5 different CNNs in predicting elevated insulin levels in cord blood based on fetal hepatic echotexture. ROC curves illustrating the performance of 5 CNNs models in predicting elevated insulin concentrations in umbilical cord blood using fetal liver echotexture. Each curve represents the model's ability to discriminate between high and normal insulin levels, with the corresponding area under the curve (AUC) values indicated.

The performance of the 5 CNN models in predicting elevated insulin levels in umbilical cord blood based on fetal hepatic echotexture is summarized in Table 3 and illustrated in Figures 5 and 6. The confusion matrices for each CNN are shown in Figure 5, while Table 3 presents the corresponding performance metrics, including sensitivity, specificity, accuracy, PPV, and NPV. Receiver operating characteristic (ROC) curves, along with the area under the curve (AUC) values for each model, are depicted in Figure 6.

**Table 3:** CNN's Performance for Identifying Neonatal Cord Blood Elevated Insulin Levels on Fetal Hepatic Ultrasound Examination in 325 Singleton Pregnancies

CNN					
Metric	ResNet 18 (%)	ResNet 34 (%)	ResNet 50 (%)	Efficient Net b0 (%)	Efficient Net b7 (%)
Sensitivity	95.8	91.7	90.4	86.5	89.4
Specificity	71.6	80.0	63.2	82.1	79.3
Accuracy	83.8	85.9	78.0	84.3	84.8
PPV	77.3	82.2	74.5	83.0	83.4
NPV	94.4	90.5	84.6	85.7	86.3

CNN, convolutional neural networks; PPV, positive predictive value; NPV, negative predictive value.

## Discussion

Current parameters for screening alterations in the fetal glycolipid profile are insufficient to adequately predict fetuses with elevated insulin levels. The identification of other non‐glycemic markers of fetal metabolic decompensation has already been encouraged in previous studies.^26^, ^27^ The contribution of AI in the medical field, particularly in diagnostic medicine, is currently growing. CNNs have been used as tools to assist in the image‐based diagnosis of tumors such as those in the breast, brain, and colon, as well as ocular and pulmonary lesions^8^, ^28^ and in the prediction of clinical events, such as in fetal medicine, where fetal lung echotexture has been correlated with respiratory distress at birth.^10^, ^11^ Fetal hyperinsulinism leads to stimulation of glycogen accumulation, increased lipid synthesis, and disproportionate growth of insulin‐sensitive tissues, including liver tissue.^29^ The affected fetal liver becomes hyperplastic and hypertrophic, which can be identified macroscopically^30^, ^31^ and by ultrasound, either through linear or volumetric measurements.^7^, ^32^ Our study represents a pioneering effort in correlating fetal hepatic imaging with neonatal insulin levels. By utilizing AI to evaluate fetal hepatic echotexture, our research has the potential to significantly improve the management of maternal‐fetal metabolic risks. This study evaluated the diagnostic performance of 5 CNNs in predicting elevated insulin levels in umbilical cord blood based on fetal liver echotexture. The comparative analysis included EfficientNet‐B0, EfficientNet‐B7, ResNet‐18, ResNet‐34, and ResNet‐50, using standard performance metrics such as sensitivity, specificity, accuracy, PPV, NPV, and the AUC. Among the models analyzed, EfficientNet‐B0 achieved the most balanced performance, with sensitivity of 86.5%, specificity of 82.1%, accuracy of 84.3%, and an AUC of 0.83. These results indicate that EfficientNet‐B0 offers a consistent ability to distinguish between normal and elevated insulin levels, with low rates of both false positives and false negatives. This profile suggests that EfficientNet‐B0 may be particularly suited for diagnostic use, where both types of misclassification can have clinical consequences. EfficientNet‐B7 showed slightly higher sensitivity (89.4%) and accuracy (84.8%) but at the cost of reduced specificity (79.3%) and a lower AUC (0.74). While EfficientNet‐B7 may be advantageous in clinical scenarios prioritizing sensitivity, such as initial screening, the increased rate of false positives may limit its utility as a definitive diagnostic tool. ResNet‐18 demonstrated the highest sensitivity (95.8%) and NPV (94.4%), minimizing the likelihood of false negatives. However, it also showed the lowest specificity (71.6%) and AUC (0.63), indicating a tendency toward overdiagnosis and limited overall discriminatory power. As such, ResNet‐18 may be more appropriate for triage applications, where ensuring that no positive case is missed outweighs concerns about false positives. ResNet‐34 achieved the highest overall accuracy (85.9%) and a well‐balanced sensitivity (91.7%) and specificity (80.0%). Although its AUC (0.69) was lower than that of EfficientNet‐B0, the model's consistent performance across all metrics suggests its potential as an alternative for clinical settings requiring both accuracy and reliability. In contrast, ResNet‐50 showed the lowest accuracy (78.0%) and specificity (63.2%), with an AUC of 0.75. Despite a high sensitivity (90.4%), the relatively high rate of false positives may limit its applicability in settings where diagnostic precision is essential.

Results from our study hold significant potential, particularly for fetuses of euglycemic mothers who present ultrasound signs of metabolic disturbances, such as macrosomia, increased abdominal circumference, and polyhydramnios. These indicators may suggest a heightened risk of neonatal high levels of insulin, even in the absence of a maternal diagnosis of gestational diabetes. Mogensen and colleagues recently highlighted the relevance of this topic, demonstrating that, even in the absence of GDM, glucose concentrations during pregnancy are positively associated with C‐peptide levels in umbilical cord blood and with the newborn's BMI. These findings suggest that fetal hyperinsulinemia and its potential consequences may occur even without maternal glycemic alterations characteristic of GDM.^5^ It is known that maternal obesity and GDM are closely intertwined, both being characterized by metabolic derangements such as hyperglycemia, inflammation, hyperinsulinemia, hyperleptinemia, and dyslipidemia in the mothers, and that their fetuses tend to exhibit higher insulin levels.^1^ Many pregnant women, with suspected macrosomia, an increased abdominal circumference, and polyhydramnios on ultrasound, are not properly counseled regarding the risks of fetal hyperinsulinemia because their glycemic profile appears normal. The absence of altered glycemic levels during pregnancy should not exclude the risk of fetal hyperinsulinemia, particularly in overweight mothers.^5^ Therefore, AI‐driven ultrasound analysis of fetal liver echotexture could provide a valuable, non‐invasive tool for identifying fetuses at increased risk of elevated insulin levels, regardless of the maternal glycemic status. Our tool could provide an objective data point that helps convince both pregnant women and their healthcare providers that, even with normal glycemic control, the fetus may still have elevated insulin levels. The potential consequences of elevated insulin levels could justify the need for lifestyle adjustments during pregnancy, ultimately enhancing prenatal care.

Although conducted at a single research center by an experienced sonographer, the methodology used to obtain fetal liver images is straightforward and reproducible. Imaging protocol employs the same fetal liver section commonly used to acquire abdominal circumference, specifically the transverse plane of the fetal abdomen. Additionally, our study utilized 3 different ultrasound machines, all with very simple preset requirements (eg, no zooming or use of calipers), which enhances the potential for scaling this test in clinical settings. This simplicity in imaging parameters contributes to the reproducibility of the technique across various centers and healthcare environments, making it a promising tool for large‐scale adoption.

Application of AI in this context opens significant opportunities for detection of metabolic risks in fetuses, which can lead to timely, targeted perinatal interventions. These interventions could include personalized neonatal follow‐up and long‐term preventive measures aimed at reducing risk of disease conditions such as non‐alcoholic fatty liver disease (NAFLD), diabetes, obesity, and cardiovascular diseases.^26^ By identifying these risks early, this technology can guide clinical decision‐making, helping to mitigate long‐term metabolic consequences associated with neonatal hyperinsulinemia.^26^

Looking forward, the impact of this technology could be substantially amplified if applied in earlier stages of pregnancy. Detecting changes in ultrasound and hepatic texture patterns early on could enable preventive interventions, promoting optimal intrauterine health. This could reduce the risk of future metabolic disorders, improving long‐term maternal and neonatal outcomes and lowering healthcare costs.

However, to validate these promising results, larger multicenter studies are needed to confirm the generalizability and applicability of this AI‐based approach. A broader sample size will provide stronger evidence regarding the effectiveness and accuracy of AI models in predicting elevated insulin levels in cord blood based on fetal hepatic texture.

Additionally, future research should focus on the long‐term outcomes of offspring with elevated insulin levels at birth. Understanding the relationship between high C‐peptide levels and the risk of metabolic complications—especially in the absence of a GDM history—is critical, as it will help identify individuals at higher risk of developing conditions such as T2DM, cardiovascular disease, and NAFLD later in life.

## Conclusion

AI‐based ultrasound texture analysis of the fetal liver accurately predicted elevated C‐peptide levels in neonatal umbilical cord blood, providing a non‐invasive method for the detection of insulin dysregulation in newborns. Among the 5 CNN architectures evaluated, EfficientNet‐B0 demonstrated the most balanced performance across key metrics, indicating its potential as the optimal model for clinical diagnostic use.

## Data Availability

The data that support the findings of this study are available from the corresponding author upon reasonable request.
